# Hydrophysical properties of sandy clay contaminated by petroleum hydrocarbon

**DOI:** 10.1007/s11356-020-07627-5

**Published:** 2020-01-10

**Authors:** Edyta Hewelke, Dariusz Gozdowski

**Affiliations:** 1 Water Centre WULS, Institute of Environmental Engineering, Warsaw University of Life Sciences, Nowoursynowska 166, 02-787 Warsaw, Poland; 2 Department of Biometry, Institute of Agriculture, Warsaw University of Life Sciences, Nowoursynowska 166, 02-787 Warsaw, Poland

**Keywords:** Soil water repellency, Petroleum hydrocarbon, Saturated hydraulic conductivity, Soil moisture retention, Surface runoff

## Abstract

The aim of the presented research was to assess the changes in hydro-physical properties of sandy clay under the influence of petroleum hydrocarbon contamination. An understanding of these changes is fundamental in the right remedial actions and for further use of soil. Laboratory tests of inherently wettable sandy clay showed that the petroleum hydrocarbon induced potential soil water repellency (SWR) of extremely repellent class at the contamination of 18 g kg^−1^. The relationship between soil water potential (pF) and SWR determined by the WDPT test for given hydrocarbon contamination, i.e., 6, 12, 18, 30, 100 g kg^−1^, showed that the critical soil moisture value (CSMC) corresponds to the pF = 1.0 ÷ 1.5. Soil retention characteristic (pF) showed that an increase in hydrocarbon contamination from 0 to 100 g kg^−1^ caused a reduction of total available water for plants from about 0.19 to 0.06 cm cm^−3^. At the same time, in the pF = 1.5 ÷ 2.0 range, intensive soil pore drainage was observed. Statistically, significant effect of hydrocarbon contamination and soil moisture potential on SWR was found. Soil hydrophobicity limits the addition of soil retention, because a significant part of the precipitation can be transformed by surface runoff. The carried out tests showed that at a hydrocarbon contamination of 30 g kg^−1^, total rainfall amount 14 mm with an intensity of 2 mm h^−1^ was transformed into a surface drain in approx. 40%. The conducted studies demonstrate the adverse impact of hydrocarbon contamination on the soil’s hydro-physical properties. The soil water retention reduction and launching of the surface outflow, as a result of limiting the water penetration process resulting from SWR, change the agrohydrological conditions of the contaminated area. It can result as the imbalance of the flow of energy and matter in the ecosystem. The scenarios of environmental effects, among others, depend on the type of soil, the degree of its pollution, the type of ecosystem, and supporting activities undertaken by man. It should be taken into account that the increasing frequency of drought occurrence associated with climate change is conducive to the phenomenon of SWR regardless of the reasons for its occurrence.

## Introduction

Contamination with petroleum hydrocarbon can occur on a large scale, due to presence of an oil rig, pipeline and tank failures, catastrophes, military conflicts, and criminal activities. A factor that is especially responsible for the visible pressure to the mass spread of petroleum hydrocarbon is floods. Contamination on a smaller scale also takes place during the normal usage of petrochemical plants, transport bases, port areas, weapons test sites, and others. The increased risk of the harmful influence on the environment and people’s heath is connected with the increased trend of using petroleum derivatives and the necessity of transporting them far distances. Contamination with petroleum hydrocarbon in Europe comprises over 50% (European Environment Agency 2012) of all registered incidents of soil contamination.

Contamination with petroleum hydrocarbon influences changes in the hydro-physical properties of soil. The dimension of these changes depends on, e.g., the type of soil, its initial properties, and the level of contamination. One of the consequences of contamination with petroleum hydrocarbon is the water repellency of soils (SWR) (Roy and McGill 1998). SWR is caused by low solid surface free energy resulting in a weak attraction between the solid particles and the liquid phase (Roy and McGill 2002). This may result in a large number of nonpolar sites on the particle surfaces (Tschapek 1984). The distribution of polar force makes the wettability of soil difficult in various degrees, and consequently, the soils are characterized by a high contact angle.

This phenomenon can have reasons of both, a natural as well as an anthropogenic nature. It is often connected with the occurrence of plant-derived waxes (e.g., Franco et al. 2000; Iovino et al. 2018; Hewelke et al. 2018a), organic carbon content (e.g., Goebel et al. 2011; Łachacz et al. 2009), organic compounds changed by fires (e.g., Granged et al. 2011; Sazawa et al. 2018). The relationship between water repellency and the manner of soil use is indicated, among others, by Urbanek et al. (2007), Zavala et al. (2009), Hejduk et al. (2017), Olorunfemi and Fasinmirin (2017), Lichner et al. (2018), and Hewelke (2019).

The effects of petroleum contamination on SWR have been studied less often. Takawira et al. (2014) indicate that on inherently wettable tropical sandy soils, hydrocarbon-induced hydrophobicity could be transient. Investigations by Li et al. (1997), Adams et al. (2008), and Gordon et al. (2018) documented that oil attenuation is not necessarily followed by a similar attenuation in the hydrophobicity as could be expected. The lack of effects from bioremediation in Li et al. (1997) studies on clay loam texture soil could be attributed to soil physical properties negatively influenced by hydrocarbon residuals due to water repellence.

Oil-contaminated soil may effect water repellency, and the persistence of SWR was much greater in the sandy soil with respect to the clayey soil in Adams et al. (2008) researches on bioremediated soils. In Gordon et al. (2018) research, the degradation of oil over 40-year time did not cause a decrease in hydrophobicity.

Research by Marín-García et al. (2016) suggested that the real risk of SWR in petroleum-contained clayey alluvial soils in Mexico occurs during the driest part of the year. Documented by Aislabie et al. (2004), hydrocarbon-contaminated soils were weakly hydrophobic in the Antarctic region, and impacts on moisture retention were negligible. The spatial variability of SWR was modulated by seasonal variations in water repellency, with the highest hydrophobicity levels occurring during the summer in the temperate continental climate zone (Buczko et al. 2006). Severe levels of SWR on soils contaminated with tar oils were largely restricted to a thin surface layer and were correlated to soil water contents.

Increased SWR decreases water sorptivity and infiltration (Vogelmann et al. 2017), which in turn increases surface runoff (Imeson et al. 1992; Cerdà et al. 2007; Miyata et al. 2007; Jordán et al. 2008), non-uniform wetting fronts with preferential flow pathways (Ritsema et al. 1993; Wallach 2010; Hewelke et al. 2014; Leuther et al. 2018), which changes the hydrological regime of the soil. This can cause erosion and the secondary spread of contaminants. Many authors indicate the seasonality of the phenomenon of water repellency, especially in a moderate climate, and its strict relationship with soil moisture content (de Jonge et al. 1999; Dekker et al. 2009; Buczko et al. 2005, 2007). Severe droughts, which have been recently and frequently occurring due to global warming, also in the moderate climate zone, are affect to water repellency. The phenomenon of water repellency also, in itself, increases the susceptibility of soil to drought as, by stopping wetting, it limits the possibilities of the ability to make use of the natural retention of soil.

Dekker and Ritsema (1994) observed that there was soil moisture content threshold above which the soil became water repellent and below which the soil was wettable. The concept of the threshold value of soil moisture content is referred as critical soil moisture content (CSMC), or critical humidity value that represents the limit between hydrophobic and wettable soil and appears rather as a threshold, but not as a sharp value (Dekker et al. 2001). Suggested by Clothier et al. (2000), Chau et al. (2014), and Hewelke et al. 2016, Hewelke 2019), the best preventive technique to minimize the development of soil water repellency was maintaining the soil above the CSMC.

Familiarity with changes which took place as a result of hydrocarbon contamination provides key information for soil remediation. According to recommendation from Adhikari and Hartemink (2016) review paper, present study directly linked change of soil properties to the soil ecosystem services (ES) limitation. The maintenance and improvement of soil ES depend on how sustainable man’s practices are (Pereira et al. 2018). The aim of the presented studies was the assessment of changes in the water properties of sandy clay contaminated with diesel. The hypothesis that hydrocarbon contamination negatively influences the water properties of the analyzed soil, which can lead to unfavorable environmental effects, was assumed.

## Materials and methods

### Experimental materials and preparations

The studied soil was derived from agricultural land located in the outskirts of Warsaw, in the area of the Kabaty underground station (N 52°12′58″ E 21°07′50″). It is currently used for growing potatoes and grains. The soil had not been previously contaminated by petroleum derivatives. The samples were collected from the top layer (horizon A, 0–10 cm). The water properties of uncontaminated soil were the reference (control) for soil properties contaminated with various doses of petroleum hydrocarbon.

### Determination of basic soil physical and chemical properties

Particle size distribution was determined using the Bouyoucos method with modifications by Casagrande and Prószyński (the areometric method) for particles lower than 0.1 mm and the sieve method for particles higher than 0.1 mm (Ryżak et al. 2009). The mass of particles oven dried at 105 °C by the volume of the core sample (in five replicates) was conducted to determine bulk density. The standard potentiometric method was used to measure soil pH in 1:5 soil water suspension. Using the Tiurin’s method (Lityński et al. 1976), organic carbon content was assessed, and using Kjeldahl’s method (Ostrowska et al. 1991), nitrogen content was determined. Triplicates were done for measurements of pH, organic carbon, and total nitrogen.

### Soil contamination preparations

A collective soil sample contained five subsamples randomly collected from the top 10-cm depth. The samples were air dried, mixed, and passed through a 2-mm sieve. Five levels of contamination were tested: 6, 12, 18, 30, and 100 g diesel per kilogram of dry soil. Similar levels of contamination had been applied in earlier studies of other authors (e.g., Adams et al. 2008; Takawira et al. 2014). The soil samples contaminated in a laboratory were subjected to a mixing process at a temperature of 60 °C in hermetical container. Next, the samples were cooled to the room temperature while mixing intensively. The stabilization processes were conducted for 14 days in the dark, at room temperature. The soil was mixed at day intervals during the aging process (Takawira et al. 2014; Wei et al. 2019).

### Evaluation of soil water repellency SWR

The SWR of each soil sample was determined using the WDPT test, which is the most widespread method (Dekker et al. 2009; Doerr et al. 2000) and is also the most suitable (Papierowska et al. 2018). The three samples of about 20 g of air dry soil from each level of contamination were placed in Petri dishes to evaluate the potential value of SWR. The five drops of distilled water from a standard medicine dropper were deposited on the gently hand-smoothed soil sample. The median values of the WDPT test corresponding to each level of contamination were used for determination of SWR classes. The assessment of SWR classes was assumed by classification proposed by Dekker and Jungerius (1990). The following five classes were distinguished: class 0, wettable, non-water repellent (infiltration within 5 s); class 1-slightly water repellent (5 s < WDPT test ≤ 60 s); class 2-strongly water repellent (60 < WDPT test ≤ 600 s); class 3-severely water repellent (600 < WDPT test ≤ 1 h); and class 4-extremely water repellent (WDPT test > 1 h). In order to establish the relationships between soil moisture content and SWR, the WDPT test was carried out for different soil moisture contents. Triplicate soil samples (100 cm^3^) for undisturbed state of control soil and for samples of contaminated soil were prepared maintaining the same bulk density of soil in its natural state. Then, samples were saturated with water by capillary rise for 3–7 days and next had been adjusted by equilibrating the material at 10 levels of soil moisture potential (pF): 0.4, 1.0, 1.5, 2.0, 2.3, 2.7, 3.0, 3.3, 3.7, and 4.2. The five drops of distilled water from a standard medicine dropper were deposited on each soil sample.

### Determination of soil hydraulic property

The reference method proposed by Klute (1986) was used to measure soil moisture retention characteristics in a laboratory on triplicate (100 cm^3^) soil samples. The moisture content values at a pF range between 0.4 and 2.0 were determined using a standard sand box, whereas the amounts of water at pF 2.3, 2.7, 3.4, and 4.2 were measured in pressure chambers. Saturated hydraulic conductivity (*K*_s_) was determined in laboratory by the constant head method. The soil samples were collected in metal cores, volume 250 cm^3^ in five replications. The saturated hydraulic conductivity was measured by laboratory permeameter made by Eijkelkamp, Agrisearch Equipment, the Netherlands; model 09.02. The samples were saturated with water from the bottom up (capillary rise) for 3–7 days prior to measuring the pF curve and K_s_ in the laboratory. Control soil was analyzed using samples in an undisturbed state, whereas samples of contaminated soil were prepared maintaining the same bulk density of soil in its natural state.

The amount of surface runoff was assessed at a laboratory test site for air dry soil, 5.5 cm layer thickness, maintaining a bulk density (ρ_s_) similar to the natural one, with a hydrocarbon contamination of 30 g kg^−1^. A rainfall with an intensity of 2 mm h^−1^ for 7 h was simulated. Surface runoff was captured by an open drain situated perpendicularly to the line of slope, which amounted to 1.5%. Water from the drain containing suspended soil particles was directed into a tank, where registration of the volume of runoff was carried out every 30 min.

## Statistical analysis

Results for selected variables were presented as means and standard deviations. Relationship between total available water for plant and hydrocarbon contamination was examined using simple nonlinear regression. Relationship between WDPT versus hydrocarbon contamination and soil water potential was evaluated using multiple polynomial regression. For all analyses, significance level was set at 0.05. The analyses were performed in Statistica 13 software.

## Results

### General soil physical and chemical properties

The analyzed 0–10-cm surface layer of soil was classified as sandy clay (Soil Survey Division Staff, 1993). The basic physical and chemical proprieties of the soil have been given in Table 1. Low soil organic carbon content (0.59%) is characteristic of the majority of mineral soils in Poland (Siebielec et al. 2017). According to agricultural criteria, the nitrogen content (0.05%) classifies the soil as poor. The C/N = 11 is similar to the average value for sandy mineral soils in Poland, i.e., C/N = 12. The pH = 6.1 classifies the soil as slightly acidic.

**Table 1:** Summary of properties of field soil samples

Characteristic	Value
Sand [%]	47
Silt [%]	49
Clay [%]	4
Soil bulk density, *n* = 5 [kg m^−3^]	1480 ± 31
Soil organic carbon, *n* = 3 [%]	0.59 ± 0.09
Nitrogen total, *n* = 3 [%]	0.054 ± 0.001
C/N	10.92
pH (H_2_0) [−]	6.1 ± 0.1

### Soil water repellency

**Fig. 1:**
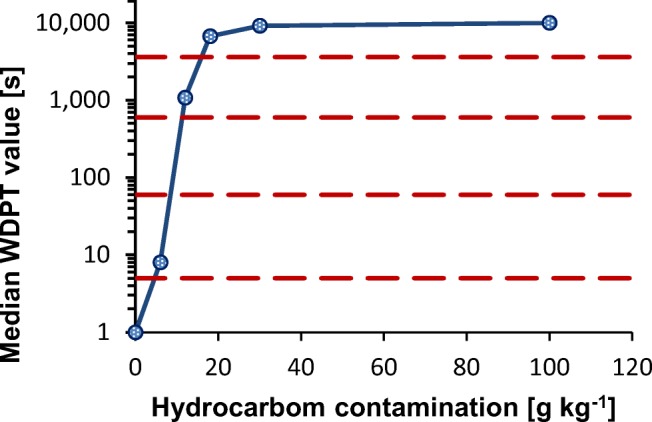
Relationship between median value of WDPT test on air dry soil—potential SWR and hydrocarbon contamination. Note: SWR classes (red dash lines): 0—wettable, non-water repellent (WDPT ≤5 s); 1—slightly water repellent (5 s < WDPT ≤ 60 s); 2—strongly water repellent (60 < WDPT ≤ 600 s); 3—severely water repellent (600 < WDPT ≤ 1 h); 4—extremely water repellent (WDPT > 1 h)

The uncontaminated soil was inherently wettable, class 0, WDPT ≤ 5 s. For a hydrocarbon contamination of 6 g kg^−1^, it reached a potential SWR value in the slightly water repellent class (class 1). For a hydrocarbon contamination of 12 g kg^−1^, the median of the WDPT value was 1080 s, which corresponds to the severely water repellent—class 3. A further increase in hydrocarbon contamination (18, 30, 100 g kg^−1^) caused a shift in the soil to the extremely water repellent class 4 (Fig. 1). Median WDPT test values between the contamination level of 30 g kg^−1^ and 100 g kg^−1^ increased from 2.55 to 2.78 h. In this range, an over threefold increase of contaminants caused a relatively small increase in the potential SWR value, i.e., by 0.23 h.

**Fig. 2:**
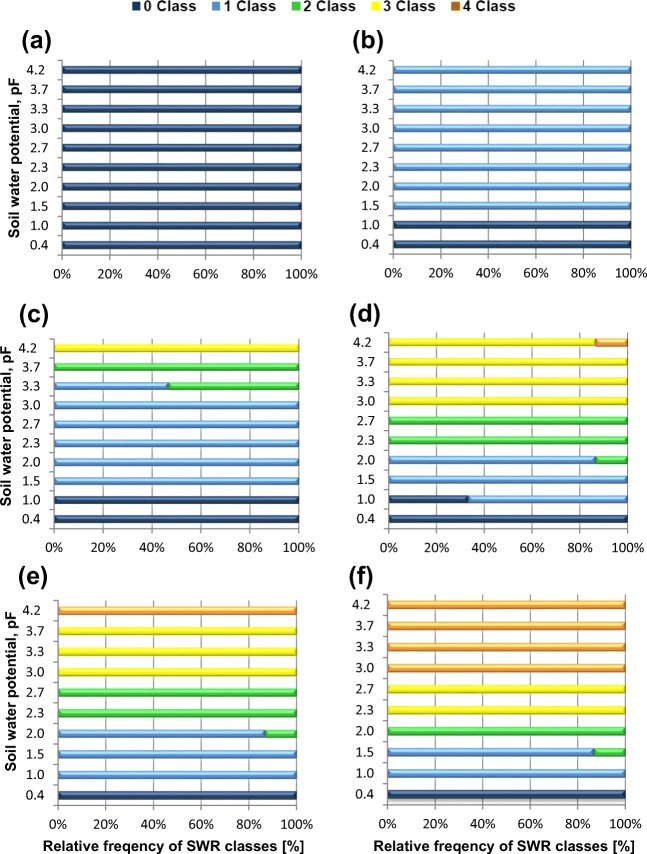
Relative frequency of SWR classes, of the A horizon of different level of hydrocarbon contamination: **a** 0 g kg^−1^; **b** 6 g kg^−1^; **c** 12 g kg^−1^; **d** 18 g kg^−1^; **e** 30 g kg^−1^; **f** 100 g kg^−1^, as a function of soil water potential in terms of pF. Note: SWR classes: 0—wettable, non-water repellent (WDPT ≤ 5 s); 1—slightly water repellent (5 s < WDPT ≤ 60 s); 2—strongly water repellent (60 < WDPT ≤ 600 s); 3—severely water repellent (600 < WDPT ≤ 1 h); 4—extremely water repellent (WDPT > 1 h)

The relationship between soil water potential and the share of individual SWR classes for each level of contamination has been presented in Fig. 2. The relationships allow for indicating the critical pF value of the given contamination at which water repellency of soil occurs. At the same time, familiarity with the retention characteristics of soil (pF curve) for the given level of contamination makes it possible to indicate CSMC as well as SWR intensity depending on moisture content. The control soil was wettable throughout the entire range of moisture content changes. With the increase in the level of hydrocarbon contamination, an increase in the frequency of occurrence of higher SWR classes was observed with a lower level of soil water potential. On the contaminated soil equal to 6 and 12 g kg^−1^, the value of CSMC corresponds to pF = 1.5. Level of hydrocarbon contaminations from 18 g kg^−1^ and higher caused a reduction of the CSMC value which corresponds to pF = 1.0.

### Saturated soil hydraulic conductivity and water retention

**Fig. 3:**
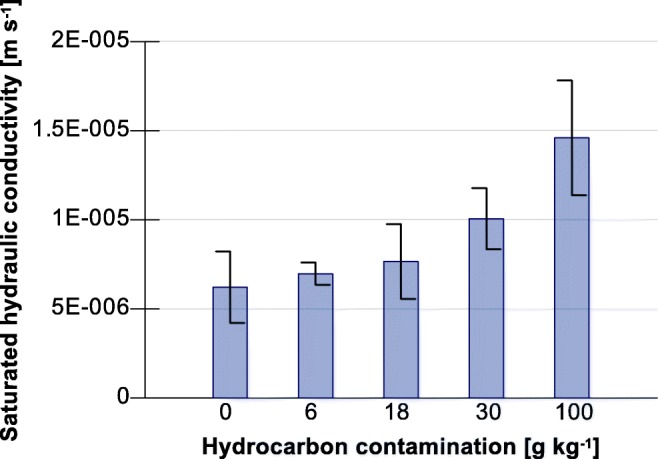
Relationship between saturated soil hydraulic conductivity and hydrocarbon contamination. Note: data are mean of five replicates, error bars show standard deviation

Saturated soil conductivity (K_s_) indicated under laboratory conditions was referred to a standard temperature of 10 °C. The average value of K_s_ for uncontaminated soil was 6.215 * 10^−6^ ± 2.002 * 10^−6^ m s^−1^. The increase in the contamination of hydrocarbon caused an upward K_s_ trend, which reached a value 1.460 * 10^−5^ ± 3.220 * 10^−6^ m s^−1^ for a contamination of 100 g kg^−1^ (Fig. 3).

**Fig. 4:**
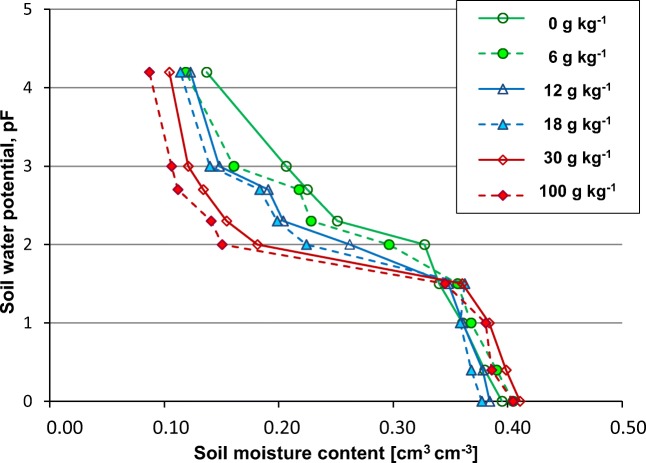
Soil moisture retention at characteristic pF levels for different hydrocarbon contamination

**Fig. 5:**
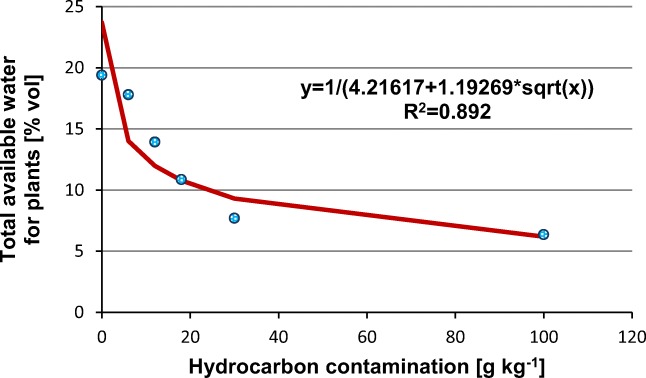
Effect of hydrocarbon contamination on total available water for plants, *P* ˂ 0.05

Soil moisture characteristic (pF) showed that hydrocarbon contamination reduced soil moisture retention (Fig. 4). Soil moisture retention in a hydrocarbon contamination range from 0 to 100 g kg^−1^ differed in terms of water availability to plants. In clean soil, field moisture content (pF = 2) was about 0.33 cm^3^ cm^−3^, whereas at a hydrocarbon contamination of 100 g kg^−1^, it was 0.15 cm^3^ cm^−3^. The wilting point of plants (pF = 4.2) in clean and contaminated soil was 0.14 and 0.09 cm^3^ cm^−3^, respectively. The total amount of water available for plants (TAW) change in the tested range of contaminations amounted to 0.13 cm^3^ cm^−3^. Particularly strong outflow of water from soil pores with ingrown contaminations was observed in the potential range pF = 1.5–2.0. The effect of petroleum hydrocarbon contamination on TAW is presented in Fig. 5. The proposed equation for TAW as a function of hydrocarbon contamination is characterized by a high determination coefficient of *R*^2^ = 0.892.

**Fig. 6:**
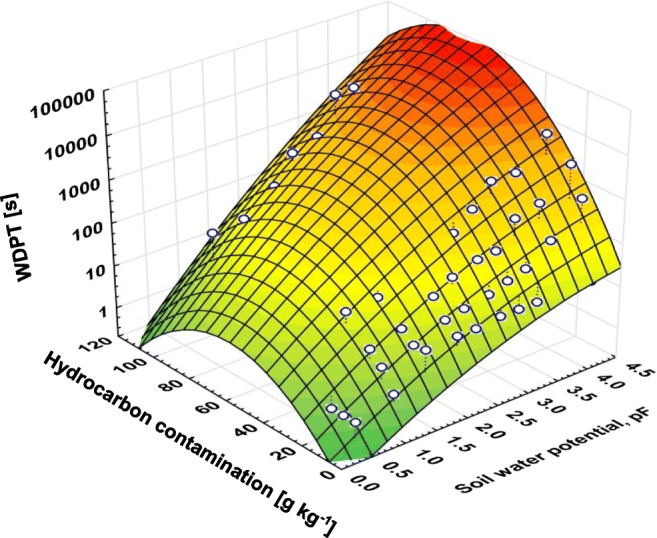
Influence of level of hydrocarbon contamination and soil water potential on soil water hydrophobicity

The impact of hydrocarbons and soil moisture content on soil hydrophobicity (Fig. 6) was expressed by the below equation:

$$ \mathit{\log}(WDPT)=-1.0915+0.9241\ast pF+0.0644\ast HC-0.0797\ast {pF}^2-0.0006\ast {HC}^2+0.0057\ast pF\ast HC $$

where

*pF*: soil water potential,
*HC*: hydrocarbon contamination [g kg^−1^].

Soil water content and level of hydrocarbon contamination statistically significantly (*P* < 0.05) influenced on SWR. The determination coefficient (*R*^2^) was 91.4%, which has proved the strong impact of the studied variables and has enabled estimation of SWR for specific values of pF and hydrocarbon contamination in the range that was assessed.

### Surface runoff

**Fig. 7:**
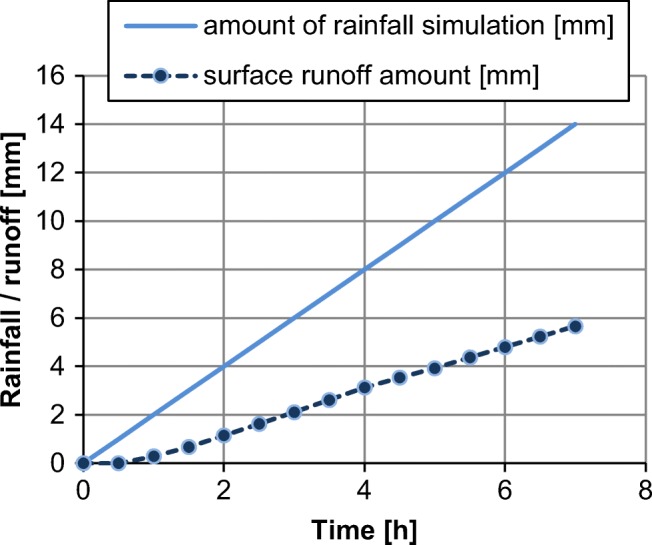
Surface runoff from sandy clay at a hydrocarbon contamination of 30 g kg^−1^, rainfall intensity of 2 mm h^−1^ with 1.5% slope of terrain

**Fig. 8:**
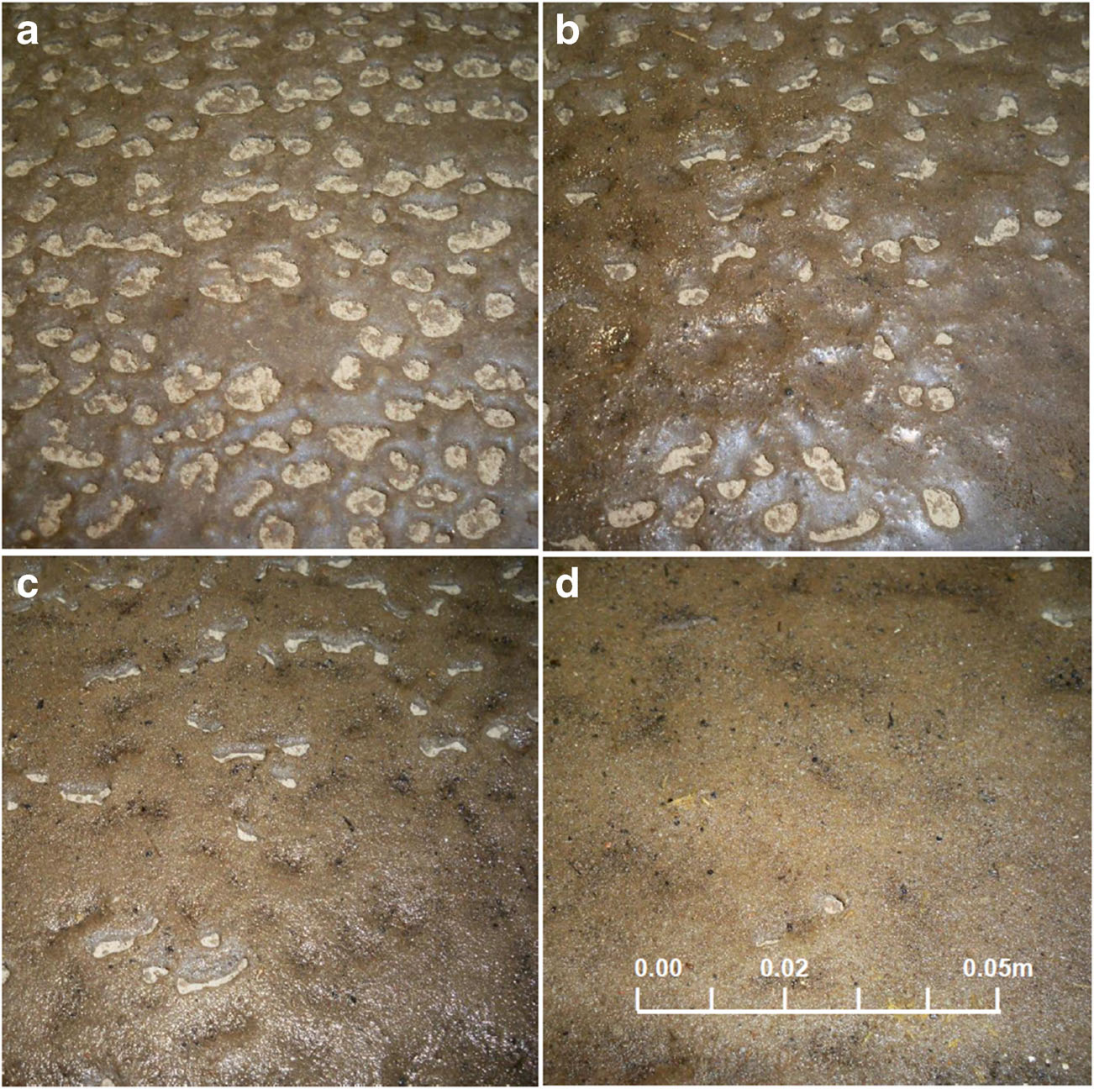
Visualization of wetted surface area over time during simulated rainfall: **a** after 1 h, **b** after 3 h, **c** after 4 h, and **d** after 5.5 h

Intensive surface runoff of part of the rainfall as well as soil erosion is often a natural consequence of water repellency. The results of the rainfall simulation were presented for a hydrocarbon contamination of 30 g kg^−1^ in Figs. 7 and 8a–d. Surface runoff amounted to 40.5% of total rainfall at soil erosion of 71.4 g m^−2^. Soil surfaces of high moisture content, as well as completely dry surfaces, are noticeable from which surface runoff takes place. The thickness of the wetted layer after completion of the experiment was approximately 3–5 mm, while the soil below was completely dry.

## Discussion

Familiarity with the hydrophysical properties of soil contaminated with petroleum hydrocarbon is of key significance when establishing the parameters of bioremediation technology. Especially important is the assessment of the water repellency of soil depending on its contamination and moisture content. According to earlier studies (Hewelke et al. 2018b) carried out on sandy soils contaminated with petroleum hydrocarbon, the fastest natural biodegradation of the contaminating substance was obtained at a moisture content close to field capacity.

The tested sandy clay (sand 47%) was characterized by a rising potential WDPT value trend depending on the level of contamination. The rising WDPT trend was also obtained by Takawira et al. (2014) for fine sandy loam (sand 80–88%). When comparing both soils, for identical hydrocarbon contaminations, significantly higher values of potential WDPT were obtained for sandy clay. The critical potential of soil water for water repellency corresponded to a CSMC of approx. 0.35 cm^3^ cm^−3^. In a humid tropical environment (Adams et al. 2008) sandy soil presented serve SWR in a relatively low petroleum contaminations, and it began much greater than in the clayey soils. For the clayey soil contaminated with 4% heavy crude, the CSMC studied by Marín-García et al. (2016) was 13.7%, while only in the driest month, in situ moisture content was observed equal to 14.83% on the soil surface and 15.50% in the cracks. Investigations provided by Buczko et al. (2006) on sandy soil contaminated with tar oils showed CSMS of about 2.5–4%. The spatial variability of soil water repellency, with the highest hydrophobicity levels occurring during the summer was correlated to critical soil water contents. Limited oil degradation due to the extremely dry conditions was observed by Stavi and Rosenzweig (2019) in Israel. In Adams et al. (2016) studies on very sandy soils, SWR, rather than toxicity, was causing the loss of vegetation, samples of 100 mg kg^−1^ petroleum hydrocarbon concentrations had severe water repellency and CSMC was directly related to hydrocarbon contamination.

The soil water retention decreased due to the reduction of soil hydrophilicity (Takawira et al. 2014; Wei et al. 2019), which was also observed in presented investigations on inherently wettable sandy clay soil. Wei et al. (2019) also emphasize that oil viscosity may have a significant impact on soil saturation. In the present study, hydrocarbon contamination significantly affected soil water retention with a pressure head greater than pF = 1.5. Above this value, an increase in pollution caused a significant reduction in soil water retention, which was also observed by Wei et al. (2019). At low pF values, the impact of contamination on soil water retention was ambiguous, probably due to the use of a relatively low viscosity oil.

Increasing water repellency limits the possibilities of retention and because a significant part of rainfall water can runoff from the surface or by preferential flows, at the same time carrying contamination over large distances. The measured significant surface runoff and soil mass, carried off with it, indicate, despite limitations posed by the laboratory experiment, that the erosion process had already been triggered at a relatively low slope of terrain. Changes in the water balance under the influence of water repellency are indicated, among others by Ritsema et al. (1993) and Wang et al. (2000), Klamerus-Iwan et al. (2015), and Buzmakov et al. (2019). In connection with the above, where environmental protection is concerned, the proper strategy for hydrophobic soils is maintaining adequately high moisture content.

Saturated soil water conductivity increased along with the contamination of hydrocarbons. The reasons behind this phenomenon in soil contaminated with hydrocarbon are connected with the fall of the dielectric constant of water. Fernandez and Quigley (1985) observed an increase in K_s_ in clay soils of 5 orders of magnitude ranging from 5 * 10^−9^ to 1 * 10^−4^ cm s^−1^, with a decline in the dielectric constant from 80 to 2. In the analyzed sandy clay, in the range of hydrocarbon contamination from 0 to 100 g kg^−1^, the increase in K_s_ was approx. eight-fold. Soil moisture curves measured for various contaminations of hydrocarbon indicate that, in the range of potential pF = 1.5–2.0, an intensive emptying of soil pores occurs. As a consequence, the total content of water available to plants was reduced from 0.19 cm ^3^ cm^−3^ for uncontaminated soil to 0.06 cm^3^ cm^−3^ for soil with a hydrocarbon contamination at a level of 100 g kg^−1^. In earlier studies, Roy and McGill (1998) and Takawira et al. (2014) obtained a similar retention reduction trend under the influence of hydrocarbon contamination. The reduction in retention observed in the analyzed case is a factor significantly increasing susceptibility to drought. Drought not only induces water repellency but it also intensifies it. Under conditions of soil drought, the natural reduction in hydrocarbons by native soil bacteria could be slowed and not very efficient (Hewelke et al. 2018b).

## Summary and conclusions

The results of studies indicate that hydrocarbon already induced potential soil water repellency (SWR) in sandy clay in the extremely repellent class at a contamination of just 18 g kg^−1^. CSMC for water repellency was observed at a soil water potential of pF ≈ 1.5. At the same time, the increase in the contamination of hydrocarbons caused a strong reduction in soil retention. The presented changes in the water properties of sandy clay have a detrimental influence on the water balance of soil, particularly increasing surface runoff. Surface runoff, usually combined with erosion, will result in the transfer of contaminants into surface waters and soil onto clean areas. A reduction in retention significantly increases susceptibility to drought and limits plant growth. Increasing the susceptibility of soil to drought causes the weakening of the activity of native soil bacteria and decreasing the natural self-repair abilities of the ecosystem. In connection with the above, it ought to be acknowledged that soil functions were severely damaged due the contamination with petroleum hydrocarbon.

## References

[CR1] Adams RH, Osorio FG, Cruz JZ (2008). Water repellency in oil contaminated sandy and clayey soils. Int J Environ Sci Technol.

[CR2] Adams RH, Cerecedo-López RA, Alejandro-Álvarez LA, Domínguez-Rodríguez VI, Nieber JL (2016). Treatment of water-repellent petroleum-contaminated soil from Bemidji, Minnesota, by alkaline desorption. Int J Environ Sci Technol.

[CR3] Adhikari K, Hartemink AE (2016). Linking soils to ecosystem services—a global review. Geoderma.

[CR4] Aislabie JM, Balks MR, Foght JM, Waterhouse EJ (2004). Hydrocarbon spills on Antarctic soils: effects and management. Environ Sci Technol.

[CR5] Buczko U, Bens O, Hüttl RF (2005). Variability of soil water repellency in sandy forest soils with different stand structure under scots pine (Pinus sylvestris) and beech (Fagus sylvatica). Geoderma.

[CR6] Buczko U, Bens O, Durner W (2006). Spatial and temporal variability of water repellency in a sandy soil contaminated with tar oil and heavy metals. J Contam Hydrol.

[CR7] Buczko U, Bens O, Hüttl RF (2007). Changes in soil water repellency in a pine–beech forest transformation chronosequence: influence of antecedent rainfall and air temperatures. Ecol Eng.

[CR8] Buzmakov S, Egorova D, Gatina E (2019). Effects of crude oil contamination on soils of the Ural region. J Soils Sediments.

[CR9] Cerdà A, Olorunfemi IE, Fasinmirin SH (2007). Soil wettability, runoff and erodibility of major dry-Mediterranean land use types on calcareous soils. Hydrol Process.

[CR10] Chau HW, Biswas A, Vujanovic V, Si BC (2014). Relationship between the severity, persistence of soil water repellency and the critical soil water content in water repellent soils. Geoderma.

[CR11] Clothier BE, Vogeler I, Magesan GN (2000). The breakdown of water repellency and solute transport through a hydrophobic soil. J Hydrol.

[CR12] de Jonge LW, Jacobsen OH, Moldrup P (1999). Soil water repellency: effects of water content, temperature, and particle size. Soil Sci Soc Am J.

[CR13] Dekker LW, Jungerius PD (1990). Water repellency in the dunes with special reference to the Netherlands. Catena.

[CR14] Dekker LW, Ritsema CJ (1994). How water moves in a water repellent sandy soil: 1. Potential and actual water repellency. Water Resour Res.

[CR15] Dekker LW, Doerr SH, Oostindie K, Ziogas AK, Ritsema CJ (2001). Water repellency and critical soil water content in a dune sand. Soil Sci Soc Am J.

[CR16] Dekker LW, Ritsema CJ, Oostindie K, Moore D, Wesseling JG (2009) Methods for determining soil water repellency on field-moist samples. Water Resour Res 45(4). 10.1029/2008WR007070

[CR17] Doerr SH, Shakesby RA, Walsh R (2000). Soil water repellency: its causes, characteristics and hydro-geomorphological significance. Earth Sci Rev.

[CR18] European Environment Agency (2012) European Environment Agency Overview of contaminants affecting soil and groundwater in Europe. Published 12 Nov 2009. Available at http://www.eea.europa.eu/data-and-maps/figures/overview-of-contaminants-affecting-soil-and-groundwater-in-europe (2012), Accessed 8th May 2019

[CR19] Fernandez F, Quigley RM (1985). Hydraulic conductivity of natural clays permeated with simple liquid hydrocarbons. Can Geotech J.

[CR20] Franco CMM, Michelsen PP, Oades JM (2000). Amelioration of water repellency: application of slow-release fertilisers to stimulate microbial breakdown of waxes. J Hydrol.

[CR21] Goebel MO, Bachmann J, Reichstein M, Janssens IA, Guggenberger G (2011). Soil water repellency and its implications for organic matter decomposition–is there a link to extreme climatic events?. Glob Chang Biol.

[CR22] Gordon G, Stavi I, Shavit U, Rosenzweig R (2018). Oil spill effects on soil hydrophobicity and related properties in a hyper-arid region. Geoderma.

[CR23] Granged AJ, Jordán A, Zavala LM, Bárcenas G (2011). Fire-induced changes in soil water repellency increased fingered flow and runoff rates following the 2004 Huelva wildfire. Hydrol Process.

[CR24] Hejduk L, Hejduk A, Baryła A, Hewelke E (2017). Influence of selected factors on erodibility in catchment scale on the basis of field investigation. J Ecol Eng.

[CR25] Hewelke E (2019). Influence of abandoning agricultural land use on Hydrophysical properties of Sandy soil. Water.

[CR26] Hewelke E, Szatyłowicz J, Gnatowski T, Oleszczuk R (2014). Spatial variability in soil moisture content under preferential flow in hydrophobic organic soil. Rocznik Ochrona Środowiska.

[CR27] Hewelke E, Szatyłowicz J, Gnatowski T, Oleszczuk R (2016). Effects of soil water repellency on moisture patterns in a degraded sapric histosol. Land Degrad Dev.

[CR28] Hewelke E, Oktaba L, Gozdowski D, Kondras M, Olejniczak I, Górska E (2018). Intensity and persistence of soil water repellency in pine forest soil in a temperate continental climate under drought conditions. Water.

[CR29] Hewelke E, Szatyłowicz J, Hewelke P, Gnatowski T, Aghalarov R (2018). The impact of diesel oil pollution on the hydrophobicity and CO 2 efflux of forest soils. Water Air Soil Pollut.

[CR30] Imeson AC, Verstraten JM, Van Mulligen EJ, Sevink J (1992). The effects of fire and water repellency on infiltration and runoff under Mediterranean type forest. Catena.

[CR31] Iovino M, Pekárová P, Hallett PD, Pekár J, Lichner Ľ, Mataix-Solera J (2018). Extent and persistence of soil water repellency induced by pines in different geographic regions. Journal of Hydrology and Hydromechanics.

[CR32] Jordán A, Martínez-Zavala L, Bellinfante N (2008). Heterogeneity in soil hydrological response from different land cover types in southern Spain. Catena.

[CR33] Klamerus-Iwan A, Błońska E, Lasota J, Kalandyk A, Waligórski P (2015). Influence of oil contamination on physical and biological properties of forest soil after chainsaw use. Water Air Soil Pollut.

[CR34] Klute A (1986) Methods of soil analysis. Part 1. Physical and mineralogical methods. Klute A Agronomy monographs. ASA and SSA, Madison, WI, USA, 9

[CR35] Łachacz A, Nitkiewicz M, Kalisz B (2009). Water repellency of post-boggy soils with a various content of organic matter. Biologia.

[CR36] Leuther F, Weller U, Wallach R, Vogel HJ (2018). Quantitative analysis of wetting front instabilities in soil caused by treated waste water irrigation. Geoderma.

[CR37] Li X, Feng Y, Sawatsky N (1997). Importance of soil-water relations in assessing the endpoint of bioremediated soils. Plant Soil.

[CR38] Lichner L, Felde VJ, Büdel B, Leue M, Gerke HH, Ellerbrock RH, Kollár J, Rodný M, Šurda P, Fodor N, Sándor R (2018). Effect of vegetation and its succession on water repellency in sandy soils. Ecohydrology.

[CR39] Lityński T, Jurkowska H, Gorlach E (1976). Chemical and agriculture analysis.

[CR40] Marín-García DC, Adams RH, Hernández-Barajas R (2016). Effect of crude petroleum on water repellency in a clayey alluvial soil. Int J Environ Sci Technol.

[CR41] Miyata S, Kosugi KI, Gomi T, Onda Y, Mizuyama T (2007). Surface runoff as affected by soil water repellency in a Japanese cypress forest. Hydrol Process Int J.

[CR42] Olorunfemi IE, Fasinmirin JT (2017). Land use management effects on soil hydrophobicity and hydraulic properties in Ekiti state, forest vegetative zone of Nigeria. Catena.

[CR43] Ostrowska A, Gawliński S, Szczubiałka Z (1991). Methods of analysis and assessment of soil and plant properties.

[CR44] Papierowska E, Matysiak W, Szatyłowicz J, Debaene G, Urbanek E, Kalisz B, Łachacz A (2018). Compatibility of methods used for soil water repellency determination for organic and organo-mineral soils. Geoderma.

[CR45] Pereira P, Bogunovic I, Muñoz-Rojas M, Brevik EC (2018). Soil ecosystem services, sustainability, valuation and management. Current Opinion in Environmental Science & Health.

[CR46] Ritsema CJ, Dekker LW, Hendrickx JMH, Hamminga W (1993). Preferential flow mechanism in a water repellent sandy soil. Water Resour Res.

[CR47] Roy JL, McGill WB (1998). Characterization of disaggregated nonwettable surface soils found at old crude oil spill sites. Can J Soil Sci.

[CR48] Roy JL, McGill WB (2002). Assessing soil water repellency using the molarity of ethanol droplet (MED) test. Soil Sci.

[CR49] Ryżak M, Bartminski P, Bieganowski A (2009). Method for determination of particle size distribution of mineral soils. Acta Agrophysica.

[CR50] Sazawa K, Yoshida H, Okusu K, Hata N, Kuramitz H (2018). Effects of forest fire on the properties of soil and humic substances extracted from forest soil in Gunma, Japan. Environ Sci Pollut Res.

[CR51] Siebielec G, Smreczak B, Klimkowicz-Pawlas A, Kowalik M, Kaczyński R, Koza P, Ukalska-Jaruga A, Łysiak M, Wójtowicz U, Poręba L (2017). Report on the third phase of the contract “monitoring of arable soil chemistry in Poland in 2015–2017”.

[CR52] Soil Survey Division Staff (1993). Soil Survey Manual.

[CR53] Stavi I, Rosenzweig R (2019) Tillage effect on hydrophobicity and hydrological properties of oil-contaminated sediments in ahyper-arid region. Arid Land Res Manag:1–10. 10.1080/15324982.2019.1599468

[CR54] Takawira A, Gwenzi W, Nyamugafata P (2014). Does hydrocarbon contamination induce water repellency and changes in hydraulic properties in inherently wettable tropical sandy soils?. Geoderma.

[CR55] Tschapek M (1984). Criteria for determining the hydrophilicity–hydrophobicity of soils. J Plant Nutr Soil Sci.

[CR56] Urbanek E, Hallett P, Feeney D, Horn R (2007). Water repellency and distribution of hydrophilic and hydrophobic compounds in soil aggregates from different tillage systems. Geoderma.

[CR57] Vogelmann ES, Reichert JM, Prevedello J, Awe GO, Cerdà A (2017). Soil moisture influences sorptivity and water repellency of topsoil aggregates in native grasslands. Geoderma.

[CR58] Wallach R (2010) Effect of soil water repellency on moisture distribution from a subsurface point source. Water Resour Res 46(8). 10.1029/2009WR007774

[CR59] Wang Z, Wu QJ, Wu L, Ritsema CJ, Dekker LW, Feyen J (2000). Effects of soil water repellency on infiltration rate and flow instability. J Hydrol.

[CR60] Wei Y, Wang Y, Han J, Cai M, Zhu K, Wang Q (2019). Analysis of water retention characteristics of oil-polluted earthy materials with different textures based on van Genuchten model. J Soils Sediments.

[CR61] Zavala LM, González FA, Jordán A (2009). Intensity and persistence of water repellency in relation to vegetation types and soil parameters in Mediterranean SW Spain. Geoderma.

